# Survey on Antimicrobial Drug Use Practices in California Preweaned Dairy Calves

**DOI:** 10.3389/fvets.2021.636670

**Published:** 2021-04-22

**Authors:** Emmanuel Okello, Deniece R. Williams, Wagdy R. ElAshmawy, Jaymes Adams, Richard V. Pereira, Terry W. Lehenbauer, Sharif S. Aly

**Affiliations:** ^1^Veterinary Medicine Teaching and Research Center, School of Veterinary Medicine, University of California, Davis, Tulare, CA, United States; ^2^Department of Population Health and Reproduction, School of Veterinary Medicine, University of California, Davis, Davis, CA, United States; ^3^Department of Internal Medicine and Infectious Diseases, Faculty of Veterinary Medicine, Cairo University, Giza, Egypt; ^4^Antimicrobial Use and Stewardship Program, California Department of Food and Agriculture, Sacramento, CA, United States

**Keywords:** antimicrobial drug use, veterinary feed directive, perception, preweaned dairy calves, California Senate Bill 27

## Abstract

The California (CA) dairy industry was surveyed in July 2017 to evaluate producers' knowledge and perceptions and antimicrobial drug (AMD) use in preweaned dairy calves following the implementation of the nationwide veterinary feed directive final rule (VFD) in January 2017 and prior to statewide implementation of CA Senate Bill (SB) 27 in January 2018. Together, these regulations require veterinary oversight for all uses of medically important antimicrobial drugs (MIADs) administered to livestock in CA. Survey questionnaire was mailed to 1,361 CA Grade A milk producing dairies and calf ranches across CA resulting in a 12% (169) response. Most respondents (83%) were aware of the VFD and SB 27 changes. Use of antibiotics was perceived as important (77%) in raising preweaned dairy calves and judicious use of antibiotics was ranked as the most important antimicrobial stewardship practice, amongst record keeping, observing withdrawal periods, having a valid Veterinarian-Client-Patient-Relationship (VCPR), and use of alternatives to antibiotics. Treating sick calves was the major indication for AMD use (90.5%); however, few producers reported use of antibiotics to control (12.7%) or prevent disease (11%). Neomycin sulfate, chlortetracycline, oxytetracycline and sulfamethazine were the most used AMD. The respondents reported a decreased use of AMD in milk (10%) and in solid feed (5%), and discontinuation of one or more AMDs used in milk (18.6%) or in solid feed (5%) post-VFD rule implementation in 2017. Most respondents reported keeping treatment records and the information recorded included date (82%), dose (44%) and route (15%) of AMD used. A few respondents reported they had initiated use of alternatives to AMDs, such as vitamins (32.6%), minerals (25.6%), herbal remedies (11.6%) and pathogen specific antibodies (7%), post-VFD. The limited changes noted in AMD use could be attributed to the short period between the implementation of the VFD and the time of the survey. Our study outcomes identified opportunities to improve AMD use practices, including record keeping and use of AMD alternatives, and provides a baseline for future evaluation of the impact of these regulatory changes, as well as guidance for the future recommendations on best practices to promote judicious AMD use.

## Introduction

Antimicrobial drugs (AMD) are important compounds used in food animal production for treatment, control, and prevention of bacterial diseases. However, use of AMD is associated with development of antimicrobial resistance (AMR) (1, 2), which is a major concern for both human and animal health worldwide. Many countries have formulated and implemented surveillance programs to monitor AMD use in food animals, as the first step toward promoting their judicious use (3–9). In the United States, cattle production leads the utilization of AMD among the different livestock species. The 2018 FDA report on domestic sales and distribution of AMD for use in cattle (dairy and beef) accounted for the highest percentage (42%) of the total species-specific sales (kg) estimates for antimicrobial drugs approved for use in food animals in the United States (10). Although the U.S. does not currently have an antimicrobial drug use report in livestock that accounts for individuals at risk of being treated and a standard body weight at treatment, such as the “defined daily dose for animals,” the annual sales and distribution report has indicated decreased consumption of AMDs among the different livestock species.

In dairy cattle, the most common use of AMD are to prevent and treat mastitis in lactating and dry cows (11, 12), and for treatment or control of enteritis and respiratory diseases in calves (13). Besides direct exposure of dairy calves to AMD for treatment and prevention purposes, additional indirect exposure occurs through feeding non-saleable (waste or hospital) milk that may contain low concentrations of drug residues (14). Waste milk is milk harvested from cows treated with intramammary, oral, or injectable antimicrobials during the withholding period when such milk cannot be sold for human consumption. Waste milk may also contain milk from recently calved cows while they transition from colostrum to normal milk secretion. The practice of feeding waste milk is widespread in the dairy industry, despite research evidence which indicates that it is associated with changes in microbial populations and increased presence of AMR bacteria in calves, compared to calves fed saleable bulk tank milk (15–19).

Understanding the ways in which AMD are used in the dairy industry and estimating the associated risks for AMR is critical for understanding hurdles encountered by the dairy industry to adopt judicious drug use practices necessary to ensure the well-being of food animals and protect both veterinary and public health (20). Previous research has shown that both veterinarians and producers play crucial roles in the use of AMD; among veterinarians, prescription decision-making habits are the major factors that influence the use of AMD (21, 22), while the major drivers for AMD use among producers included the type of cattle operation, disease and animal welfare, economic factors, veterinary consultation, producer's experience and peer support, perceived drug efficacy and drug use regulations (23). Furthermore, raising food animals in a production setting sustainably should employ preventative management practices that modify the environment and host to reduce the risk of disease as a priority before using AMD. Specifically, these preventative strategies should include modifying the environment to reduce stress using proper housing, optimum colostrum management and nutrition, and use of effective vaccines (24, 25). An approach that allows producers to raise and manage food animals sustainably with emphasis on addressing the environment and host factors includes the risk assessment tool for bovine respiratory disease (26).

In the United States, recent regulatory changes were made by the Food and Drugs Administration (FDA) to improve the regulatory oversight of Veterinary Feeds Directive (VFD) drugs while continuing to protect human and animal health. The VFD drugs refer to new animal drugs intended for use in or on animal feed which are limited to use under the professional supervision of a licensed veterinarian (27). The VFD final rule was implemented effective January 1, 2017. Locally in California, Senate Bill (SB) 27 was approved and passed in 2015 as the Livestock: Use of Antimicrobial Drugs Law (California Food and Agriculture Code Sections 14400-14408) (28), here onwards referred to as SB 27. Effective January 1, 2018, SB 27 regulations restricted all uses of medically important antimicrobial drugs (MIADs) to veterinary prescription or VFD only. The term MIADs refers to antimicrobial drugs listed in Appendix A of the federal Food and Drug Administration's Guidance for Industry #152, and include critically important, highly important, and important antimicrobial drugs such as norfloxacin, cephalexin, cefaclor, penicillin, oxacillin, ampicillin, streptomycin, erythromycin, clarithromycin, tetracyclines, vancomycin, chloramphenicol and trimeth/sulfameth (29). Jointly, these regulatory changes increased veterinary oversight in the distribution and use of MIADs in livestock by changing the availability of MIADs from over-the-counter (OTC) to prescription or VFD only.

The purpose of the statewide survey described here was to document AMD use practices for preweaned dairy calves, evaluate producers' knowledge and perception on AMD use, and document any changes in management and AMD use practices following the implementation of VFD rule and prior to the implementation SB-27. In California, newborn dairy calves are either raised on-site at their source dairy or off-site in a calf nursery, commonly known as calf ranches (30). Dairies and calf ranches raise replacement dairy heifers or bulls for dairy beef. The survey targeted both dairies and calf ranches that were raising preweaned calves. The outcome of this survey furthers our understanding of the industry's AMD use practices and perceptions and provides baseline data to guide future evaluation of the rule changes as well as recommendations on best practices to promote judicious use of AMDs.

## Materials and Methods

### Study Design

A survey of the California dairy producers was conducted in July 2017. The questionnaire was pre-tested by the coauthors and several collaborators with in-depth knowledge of the CA dairy industry. The survey questionnaire was comprised of 54 questions grouped into four sections (Supplementary Material). Section 1 included questions on the respondent's role on the farm and herd demographics including location, herd size, cow breeds raised and participation in welfare audit programs; Section 2 was comprised of questions about preweaned calf management practices including housing types, feeding practices, health management protocols such as adding AMD in feed, milk or water and vaccination; Section 3 questions sought information on AMD use in preweaned calves including information sources and decision making on AMD purchased and used on the farm, availability and use of treatment protocols including dose estimation and extra-label AMD use, drugs and health records system, choice of AMD used to treat sick calves, and the extent of veterinarian involvement in on-farm AMD use practices. Section 4 assessed the knowledge and perceptions on AMD stewardship practices as well as awareness of regulations. The study materials were reviewed by the University of California, Davis, Institutional Review Board and granted IRB review exemption approval (IRB ID: 1709653-1).

### Surveys and Data Collection

The survey questionnaire was mailed to 1,361 licensed Grade A milk producing dairies and calf ranches in California. In the United States, Grade A milk is the category of milk produced under higher farm sanitary conditions (compared to Grade B) to qualify for sale or consumption as fluid beverage (31). A sample size was not conducted prior to the survey. However, a *post-hoc* sample size and power analysis based on the question on respondent knowledge of AMD regulatory changes was estimated. The mailed survey included a cover letter, questionnaire, an additional form for producers to share their comments about AMD use in preweaned calves or other dairy cattle, a follow up request form, and postage-paid return envelope. The cover letter also provided a web link to the online version of the survey as an alternate response option. Dairies and calf ranches identified to receive the survey were each assigned a confidential index number used only to identify respondents for a second mailing if they had not responded to the first mailing, and to verify the county location of their premises. The second mailing was 4 weeks after the initial mailing and was sent to addresses that did not respond the first time. Similarly, a reminder card was sent 2-weeks after each mailing to those who had not responded at the time of mailing.

### Statistical Analyses

#### Descriptive Statistics

Survey data were analyzed using Stata IC 16 (Stata Corp LLC, College Station, TX USA) and (32). Responses to questions were summarized using descriptive statistics and reported as proportions for categorical variables or means for continuous variables. Uncertainty measures including standard errors and 95% Confidence Intervals (CI) were also reported.

#### Multiple Factor Analysis

Multiple Factor Analysis (MFA), an extension of the Principle Components Analysis (PCA) was used to explain the variability of the producers responses to the survey on AMD use in preweaned calves on California dairies (33). Principle Components Analysis was achieved through dimensionality reduction of the dataset's variables, both quantitative and categorical (34), through the FactoMineR package in R (35). The MFA was performed based on a subset of 66 variables classified into 12 groups, 11 qualitative groups (65 categorical variables) and one quantitative group (one continuous variables). The first three dimensions of the MFA were used to interpret the percentage of the explained dataset's variance explained. Groups with variance of 0.4 or greater on any of the first three principal components dimensions were retained for interpretation, and within each group, variables with correlation coefficients (coordinates) of 0.4 or greater were retained for interpretation of variability.

## Results

### Respondents and Their Herd Characteristics

A *post-hoc* sample size based on the question on respondent knowledge of AMD regulations showed that a sample size of 136 respondents is needed assuming that 50% answer yes, 5% level of significance and 80% power in a two-way test, and a 10% response rate out of a total of 1,361 mailed surveys. The power analysis based on the 139 surveys returned and 115 responses indicating knowledge of AMD regulations (82.7%) resulted in a *P*-value of 0.04 and power >99%.

Of the 1,361 mailed surveys, 169 (12%) responses were received including five responses completed online. Among the 169 responses received, 23 were excluded from the analysis either because they were not completed but returned or the premise responded twice, in which case only the first response was considered. Because some respondents did not answer all the questions of the surveys included in the analysis (*n* = 146), each question was analyzed based on the number of respondents (*n*) who answered the specific question. During analysis, the respondents' locations were categorized as one of the three milk shed regions of California; Northern California (NCA), Northern San Joaquin Valley (NSJV) and Greater Southern California (GSCA), with the latter being a merger of dairies in the Southern San Joaquin Valley and the limited number of dairies in Southern California (36). Most of the respondents (84 responses; 49.7%) were from GSCA, followed by NSJV (58 responses; 34.2%) and lastly NCA (27; 16%). Among the responses received, five were from calf ranches located in the GSCA. Most of the dairy survey respondents were dairy owners (83.8%, *n* = 146), and only a smaller proportion were individuals with various roles on the dairy such as herd manager (32%, *n* = 146), calf manager (12.2%, *n* = 146) and/or calf feeder (6.2%, *n* = 146). Responses from certified organic dairy farms (8.9%, *n* = 146) were excluded from analyses on AMD use, except for the questions on availability and type of VCPR. The mean calf herd size of respondents was 246 preweaned calves, with the predominant breeds being Holsteins (79.2%). Table 1 summarizes the demographics of the respondent dairies.

**Table 1:** Summary dairy characteristics.

**Dairy characteristic**	**Mean**	**SE**	***N***	**95% Confidence limits**	
				**Lower**	**Upper**
**Herd size**					
Preweaned calves	246	43.02	67	160	331.78
Milking cows	1,507	105.12	144	1,299	1,715
Rolling herd average milk production (kg)	11,118	187.07	139	10,748.86	11,488.67
Bulk tank somatic cell count (cells/mL)	245,863	6,703	139	232,608	259,118
**Predominant breed (%)**					
Holstein	79.21	3.03	142	73.22	85.19
Jersey	11.82	2.27	142	7.34	16.3
Crossbred	7.58	1.87	142	3.88	11.28
Other	0.99	0.35	142	0.05	1.43

*The values indicate mean properties of the herds based on the participant responses to the 2017 mail and online survey of California Grade A dairy milk producers on antimicrobial drug use in preweaned dairy calves*.

### Knowledge, Perception, and Impact of VFD and SB 27 Regulatory Changes

#### Knowledge on Regulatory Changes

Most of the producers were aware of the VFD rule changes and the then soon-to-be-implemented SB 27. A total of 82.7% (*n* = 139) respondents reported knowledge of the requirement for veterinary prescription for all OTC AMD starting January 1, 2018. This regulatory change was projected by the respondents to affect the producers who were reportedly using OTC AMD according to label (35.7%, *n* = 129) or extra-label (6.6%, *n* = 129). Among the calf ranches, 3 out of the 5 respondents reported knowledge of regulatory changes that were set to start on January 1, 2018 at the time of the survey, and one respondent reported using OTC AMD according to label.

#### Knowledge and Perception of Antimicrobial Stewardship Practices

The producers' knowledge and perceptions of AMD stewardship practices were assessed by asking respondents to rank the relative importance of five key stewardship practices ranging from most important (1) to least important (5). The responses for each of these practices were then ranked in order based on their mean values. Respondents ranked administration of appropriate AMD, dose, route and duration as the most important (1st), followed by good record keeping (2nd), observing withdrawal periods and drug residue avoidance (3rd), having a current veterinary-client-patient-relationship (VCPR) (4th), and the least important was the use of alternatives to antibiotics (5th). Details of the ranking are summarized in Table 2. The same ranking order was observed among the 5 calf ranch respondents.

**Table 2:** Ranking of antimicrobial stewardship practices by survey respondents.

**Stewardship practices**	**Mean**	**SE**	***N***	**95% Confidence limits**	
				**Lower**	**Upper**
Appropriate drug, dose, route, duration	1.91	0.1	134	1.72	2.1
Good record keeping	2.65	0.11	132	2.44	2.86
Observing withdrawal periods	2.73	0.12	131	2.50	2.97
Having current VCPR^*^	3.03	0.13	133	2.78	3.28
Alternatives to antimicrobial drugs	3.69	0.14	131	3.41	3.96

*The five stewardship practices were ranked from 1 (most important) to 5 (least important) based on the respondent's perception. The mean rank for each stewardship practice was calculated based on all the responses by respondents*.

* *VCPR is veterinarian-client-patient relationship*.

#### Perception of Antimicrobial Drugs

The respondents perceived the use of AMD to be extremely important (39%; *n* = 64; CI: 27.67, 51.78) or important (37.5%; *n* = 64; CI: 26.29,50.23) in raising preweaned dairy calves, and only a small percentage indicated that AMD were somewhat important (17.19%; *n* = 64; CI: 9.62, 28.8) or not important (10.9%; *n* = 64; CI: 5.2, 21.58). In the calf ranch responses, AMD use was perceived by respondents as extremely important (two respondents), important (one respondent) or somewhat important (two respondents). The importance of AMD in raising preweaned dairy calves was further emphasized by the fact that 62.5% (*n* = 65; CI: 49.77, 73.71) of respondent dairies predicted increased disease prevalence if AMD use for dairy calves was ceased completely. Similarly, 73.4% (*n* = 64; CI: 60.99, 83.02) and 54.7% (*n* = 64; CI: 42.12, 66.81) of respondent dairies predicted poor animal welfare and decreased performance, respectively, if the use of AMD in preweaned calves was stopped. Only a small proportion of the respondents indicated there would be no effect if AMD use in preweaned calves was stopped (14.1%; *n* = 64; CI: 7.39, 25.24) or were organic producers who were not using antibiotics (7.8%; *n* = 64; CI: 3.21, 17.8). The same response pattern was seen among calf ranches with four in five respondents predicting increased disease prevalence, poor growth, and compromised animal welfare, and only one respondent predicting no effect.

#### Change in Cost of Antimicrobials

Cost-wise, most of the respondents reported no change in the cost of AMD in their operations following the implementation of VFD regulatory changes (69.7%; *n* = 59; CI: 56.28, 80.12). Majority of the respondents reported no change in the cost of antibiotics following the implementation of VFD changes (70.5%; *n* = 61; CI: 56.28, 80.12), while only a small proportion of the respondents indicated that the cost of AMD had either increased (13.1%; *n* = 61; CI: 6.56, 24.49) or decreased (16.4%; *n* = 61; CI: 8.89, 28.26). Two calf ranch respondents reported an increase in cost of AMD while three respondents reported a decrease in cost.

#### Change in Antimicrobial Drug Use

Most of the producers reported no change in the use of AMD in milk (61%, *n* = 59), solid feed (81.7%, *n* = 60) or water (8.6%, *n* = 61) for preweaned calves. The details of the specific changes reported in AMD use are summarized in Table 3. A small proportion of respondents (10.9%; *n* = 55) reported a decrease in use of OTC AMD labeled for feed that do not fall under VFD requirements (e.g., amprolium); this decrease was mainly due to reduced use of these AMDs in milk. Respondents that reported a decrease in OTC AMD use also reported an increase in calf mortality. A single respondent (1.9%, *n* = 54) reported increased use of OTC AMD labeled for feed that do not fall under the VFD, explaining that reduced use of OTC AMD in milk “resulted in more aggressive use through other routes.” Some respondents reported they had initiated use of alternatives to AMD after the VFD requirements were implemented such as herbal remedies (11.6%, *n* = 43) and pathogen specific antibodies derived from sources such as eggs (7%, *n* = 43).

**Table 3:** Changes in the use of antimicrobial drugs in preweaned calves on California dairies following the implementation of the Veterinary Feed Directive (VFD) final rule on January 1, 2017 compared to practices during the year 2016.

**Changes since Jan 1st 2017**	**Percent**	**SE**	***N***	**95% Confidence limits**	
				**Lower**	**Upper**
**Antimicrobial drugs used in milk or milk replacer**					
No changes made	61.0	6.4	59	47.7	72.9
Increased amount or duration	1.7	1.7	59	0.2	11.7
Decreased amount or duration	10.1	4.	59	4.5	21.3
Discontinued 1 or more	18.6	5.1	59	10.5	31
Added 1 or more	1.7	1.7	59	0.2	11.7
Other^a,b^	8.4	3.7	59	3.5	19.2
**Antimicrobial drugs used in solid feed**					
No changes made	81.7	5	60	69.4	89.7
Increased amount or duration	0		60		
Decreased amount or duration	5	2.8	60	1.6	14.8
Discontinued 1 or more	5	2.8	60	1.6	14.8
Added 1 or more	1.7	1.7	60	0.2	11.5
Other^a^	5	2.8	60	1.6	14.8
**Antimicrobial drugs used in water**					
No changes made	83.6	4.8	61	71.7	91.1
Increased amount or duration	0		61		
Decreased amount or duration	1.6	1.6	61	0.2	11.3
Discontinued 1 or more	1.6	1.6	61	0.2	11.3
Added 1 or more	4.9	2.8	61	1.5	14.6
Other^a^	6.6	3.2	61	2.4	1.7

a *Used antimicrobial drugs occasionally*.

b *Stopped using uniprim (trimethoprim and sulfadiazine oral antibiotic powder)*.

### Antimicrobial Drug Use Practices in Preweaned Dairy Calves

Preweaned dairy calves were reportedly exposed directly to AMD for treatment, control, or prevention purposes through parenteral or oral administration, or by adding in milk, solid feed, or water. Indirect exposure to AMDs reportedly occurred through feeding non-saleable or waste milk containing drug residues. Sources of non-saleable milk included recently calved cows that were previously treated with long acting intramammary AMD infusion at dry-off, mastitis cows treated with intramammary or other parenteral AMD or other lactating cows treated with systemic AMD as treatment for other health conditions.

#### Exposure of Preweaned Dairy Calves to Antimicrobial Drugs

Indirect exposure of preweaned dairy calves to AMD occurred mainly through feeding of milk sources that presumably contained AMD residues. The mean proportion of liquid diet fed to calves by the responding dairies included non-saleable or waste milk (44.2%; *n* = 68; CI: 34.34, 53.96), saleable or bulk tank milk (28.31%; *n* = 68; CI: 18.73, 37.89), milk replacer (20.56%; *n* = 68; CI: 12.8, 28.32), and other minor sources such as transition cow milk, fortified non-saleable milk and non-fat dry milk powder (3.94%; *n* = 68; CI: −0.21, 8.09). Sources of milk containing AMD residues could have included waste (hospital) milk, as well as colostrum and milk from transition cows treated at dry-off with long acting intramammary (IMM) AMD. Most of the responding dairies treated all cows (77.5%; *n* = 142; CI: 69.77, 83.66) while a few dairies treated cows selectively (4.9%; *n* = 142; CI: 2.35, 10.06) at dry-off with long acting IMM AMD. Dairy reported sources of colostrum fed to calves included pooled colostrum (51.2%; *n* = 141; CI: 43.74, 58.6), individual cow colostrum (34.6%; *n* = 142; CI: 27.47, 41.74) or direct nursing from the dam (5.5%; *n* = 139; CI: 2.28, 8.65). A few respondents reported feeding transition cow milk to preweaned calves 5.42%; *n* = 48; CI: 0.88, 10). Among the calf ranches, 2 out of 5 respondents reported feeding preweaned calves with a liquid diet comprised of either 75 or 100% saleable milk.

Direct exposure of preweaned dairy calves to AMD occurred parenterally or orally in milk, grain, or water. More than half of the respondents (64%; *n* = 61) had a treatment protocol developed by either a veterinarian (50%, *n* = 34), farm owner (17.6%, *n* = 34) or both veterinarian and owner (30.3%, *n* = 33). The availability, content and access to the treatment protocols are summarized in Table 4. Only 27.8% (*n* = 61) respondents reported submission of calves for diagnosis, while 30.2% (*n* = 61) used other diagnostic techniques to guide antimicrobial treatment of preweaned calves. Treatment of calves using AMD was reported to mainly follow label-use (78.3%, *n* = 60) and only a small proportion of respondents reported extra-label use (16.7%, *n* = 60) or did not know if antibiotics were being used extra-label (5%, *n* = 60). Generally, most of the respondents reported following label recommendations when estimating treatment dosage (87.1%, *n* = 62) and treatment duration for both parenteral or oral AMD administration (88.1%, *n* = 59) or AMDs added in feed (74.6%, *n* = 55). The major indications for AMD use in calves, methods for estimating treatment dosage and treatment duration are summarized in Table 5. Individual treatment of sick calves was the single most important indication for AMD use (90.5%, *n* = 63), whereas use for control of ongoing diseases (12.7%, *n* = 63) or prevent disease in high-risk calves (11.1%, *n* = 63) were minor indications. Table 6 shows the mean percentage of calves that received different AMD administrations between birth and weaning. Neomycin and oxytetracycline were the most used AMDs administered to more than half of the calves during the preweaning period. The list of common AMDs of choice for treating respiratory diseases and diarrhea or enteritis in preweaned calves is shown in Table 7. The most common antimicrobials reportedly used by respondents as first choice treatment for respiratory disease and enteritis were florfenicol (43% respondents) and sulfonamide (24.4% respondents), respectively.

**Table 4:** Use of treatment protocols for health management in preweaned calves on California dairies.

**Treatment protocol (presence, content, access, development, and review)**	**Percent**	**SE**	N^*^	**95% CI**	
				**Lower**	**Upper**
**Availability and author of written treatment protocols**					
Dairies with written or computerized treatment protocol	63.9	62	61	50.9	75.2
Protocol developed by veterinarian	50	8.7	34	33	67
Protocol developed by owner	17.7	6.6	34	7.8	35.2
Protocol developed by owner and veterinarian	30.3	8.1	33	16.6	48.8
**Information contained in treatment protocols**					
Disease definitions	41	8	39	26.3	57.6
Disease specific treatments	66.7	7.7	39	49.9	80.1
**Antimicrobial drug use information contained in treatment protocols**					
Dosage	64.1	7.8	39	47.4	78
Duration	59	79.8	39	42.4	73.7
Withdrawal period	51.3	8.1	39	35.3	67
Not sure of details	0				
Other information	5.1	3.6	39	1.2	19.3
**Personnel access to protocols**					
Owner	79	6.7	38	62.4	89.5
Herd manager	57.9	8.1	38	41.2	73
Office staff	5.3	3.7	38	1.2	19.8
Calf manager	57.9	8.1	38	41.2	73
Calf feeder	36.8	7.9	38	22.6	53.8
Nutritionist	10.5	5.1	38	3.82	25.8
Veterinarian	57.9	8.1	38	41.2	73
Calf treatment crew	21.1	6.7	38	10.5	37.6
**Schedule for review of treatment protocols**					
Once to twice a year	51.4	8.33	37	34.9	67.5
Every few years	18.9	6.5	37	9	35.6
I don't know	8.1	4.6	37	2.5	23.3
Other^a^	24.3	7.15	37	12.8	41.4

*The dairies were surveyed for the availability of written or computerized treatment protocols, content, and employee access to treatment protocols*.

a *Every month, when needed or when a problem occurs*.

* *A total of 61 respondents answered the question on availability and author of written treatment protocols and the rest of the responses are subset of this group*.

**Table 5:** Indications, estimation of dosage and treatment duration for antimicrobial drugs administered to preweaned dairy calves on California dairies.

**Antimicrobial drug use in preweaned calves**	**Percent**	**SE**	***N***	**95% Confidence limits**	
				**Lower**	**Upper**
Indication for antimicrobial drug use^*^					
Treat sick animals	90.5	3.7	63	80	95.7
Control ongoing disease	12.7	4.2	63	6.4	23.8
Prevent disease in high-risk calves	11.1	4.0	63	5.3	21.9
Other^a^	4.8	2.7	63	1.5	14.2
Estimating dosage^*^					
Body weight and label dosage	87.1	4.3	62	75.9	93.5
Body weight and experience	3.2	2.3	62	0.8	12.4
Body weight and vet authorization	14.5	4.5	62	7.6	26
Standard dosage by animal category	17.4	4.9	62	9.9	29.7
Level of clinical illness	3.2	2.3	62	0.8	12.4
Different approaches for different drugs	14.5	4.5	62	7.6	26
Other^b^	3.2	2.3	62	0.8	12.4
Estimating treatment duration^*^					
**a) Antimicrobial drugs added to feed**					
Follow label instruction	74.6	5.9	55	61.0	84.6
Stop early if animal is cured	20	5.4	55	33.1	-
Extend use if animal still sick	21.8	5.6	55	12.6	35.1
Based on previous results on the farm	16.4	5.0	55	8.6	29.0
Different approaches for different diseases	7.3	3.5	55	2.7	18.3
Other^c^	18.2	5.3	55	9.9	31.1
b) Antimicrobial drugs administered via injection or orally^*^					
Follow label instruction	88.1	4.3	59	76.7	94.4
Stop early if animal is cured	25.4	5.7	59	15.7	38.4
Extend use if animal still sick	35.6	6.3	59	24.2	48.9
Based on previous results on the farm	13.6	4.5	59	6.8	25.3
Different approaches for different diseases	11.9	4.3	59	5.6	23.3
Other^d^	6.8	3.3	59	17.1	-

*N, number of respondents who answered the specific questions*.

* *Some respondents chose more than one option*.

a *Antimicrobial drugs were not used, rarely used or there were no sick calves*.

b *Other methods, not specified*.

c *Use low levels continuously, do not use AMD, follow veterinarian recommendation, use AMD occasionally*.

d *Follow veterinarian recommendation or do not use AMD*.

**Table 6:** Mean percentage of preweaned dairy calves receiving antimicrobial therapy between birth and weaning with different antimicrobial drugs.

**Antimicrobial drug**	**Mean**	**SE**	***N***	**95% CI**	
				**Lower**	**Upper**
**Liquid feed (milk or milk replacer)**					
Neomycin sulfate	62.8	15.2	10	28.4	97.1
Chlortetracycline	44	22.9	5	0	100
Neomycin-oxytetracycline	36.3	21.5	4	0	100
Spectinomycin	27.5	22.5	2	0	100
Oxytetracycline	56.7	23.3	3	0	100
Sulfamethazine	29.2	14.6	6	0	66.3
Coccidiostats	100	0	4	–	–
Other	100	–	1		
**Solid feed (grain)**					
Chlortetracycline	52.5	47.5	2	0	100
Neomycin-oxytetracycline	10	–	1	–	–
Oxytetracycline	70	30	3	0	100
Sulfamethazine	5	–	1	–	–
Coccidiostats	95.6	3.29	16	88.6	100
**Water**					
Neomycin sulfate	5	–	1	–	–
Chlortetracycline	7.5	2.5	2	0	39.3
Neomycin-oxytetracycline	100	0		–	–
Spectinomycin	50	–	1	–	–
Oxytetracycline	5		1		
Sulfamethazine	75	25	2	0	100
Bacitracin	100	–	1	–	–
Coccidiostats	51.7	17.4	6	7.1	96.3
Other	5.5	4.5	2	0	62.7

*The mean percentage was calculated for number of responses (N) for a given antimicrobial compound*.

**Table 7:** Antimicrobial drugs used to treat respiratory disease and diarrhea in preweaned calves after January 1, 2017 on California dairies.

**First choice antimicrobial drug**			**Second choice antimicrobial drug**		
**Respiratory disease (*****n*** **=** **49)**	**Number of respondents**	**Percent**	**Respiratory disease (*****n*** **=** **33)**	**Number of respondents**	**Percent**
Ampicillin	1	2.0	Ampicillin	1	3.0
Ceftiofur	8	16.3	Enrofloxacin	5	15.2
Enrofloxacin	4	8.2	Enrofloxacin and Flor^c^	2	6.1
Florfenicol	21	42.9	Florfenicol	8	24.2
Gamithromycin	2	4.1	Oxytetracycline	2	6.1
Oxtetracycline	4	8.2	Penicillin	2	6.1
Penicillin	1	2.0	Tildipirosin	1	3.0
Tildipirosin	1	2.0	Tilmicosin	1	3.0
Tulathromycin	5	10.2	Tulathromycin	11	33.3
Tylosin	1	2.0			
None^*^	1	2.0			
**Diarrhea (*****n*** **=** **45)**			**Diarrhea (*****n*** **=** **17)**		
Adsorbent^*^	1	2.2	Ceftiofur	3	17.7
Ampicillin	4	8.9	Enrofloxacin	2	11.8
Ceftiofur	3	6.7	Florfenicol	1	5.9
Enrofloxacin	1	2.2	Oxytetracycline	2	11.8
Florfenicol	1	2.2	Penicillin	2	11.8
Neomycin	2	4.4	Sulfonamide	3	17.7
Oxytetracycline	1	2.2	Tulathromycin	1	5.9
Penicillin	1	2.2	Penicillin and Ceftiofur	1	5.9
SMZ/Bismuth/Charcoal^*^	1	2.2	Ampicillin	1	5.9
Salt solutions^*^	3	6.7	Others^b^^,^^*^	1	5.9
Spectinomycin	2	4.4			
Sulfamethoxazole	1	2.2			
Sulfonamide and Ceftiofur	1	2.2			
Sulfonamide	11	24.4			
Others^a,b*^	12	26.7			

*The first and second choice antimicrobial drugs (AMD) used to treat respiratory disease and diarrhea are shown as number of respondents who answered that they used this specific AMD*.

a *Bismuth subsalicylate*,

b *ivermectin*,

c *Enrofloxacin and Florfenicol*.

* *Non-antimicrobial compounds*.

#### Drug and Treatment Records

Only 32.31% (*n* = 65; CI: 21.86, 44.88) of the respondents kept a drug inventory log on the dairy, but respondents recorded AMD treatment information such as treatment date (82.3%; *n* = 62; CI: 70.34, 90.06), dose (43.6%; *n* = 62; CI: 31.52,56.37), and route (14.5%; *n* = 62; CI: 7.59, 25.99) of administration. Forty percent (*n* = 60, CI: 28.15, 53.15) of the respondents reportedly did not track the antibiotic withdrawal interval in treated calves. The record systems used by respondents who tracked AMD withdrawal, included paper records (31%; *n* = 58; CI: 20.23, 44.39), computer software such as DC 305® (Valley Ag Software, Tulare, CA 93274) (22.4%; *n* = 58; CI: 13.26, 35.3), marking the calf hutch (12.1%; *n* = 58; CI: 5.73,23.65), memory (8.6%; *n* = 58; CI: 3.54, 19.53), or used other methods including chalk and phone (3.6%; *n* = 56; CI: 0.86, 13.71). Besides withdrawal period, other drug related information tracked included drug name (60%; *n* = 60; CI: 46.85, 71.85) quantity at hand (56%; *n* = 59; CI: 42.78, 68.31), supplier (22%; *n* = 59; CI: 13.04, 34.76), expiration date (33%; *n* = 60; 22.34, 46.49), cost (27%; *n* = 59; CI: 170.8, 40.19), manufacturer (22%; *n* = 60; 12.81, 34.24), and purchase date (15%; *n* = 59; CI: 7.97, 27.21).

#### Veterinarian Client Patient Relationship (VCPR) and Antimicrobial Drug Use

Most of the respondents had a VCPR with a practicing veterinarian (89.2%; *n* = 65; CI: 78.73,94.88) and a minor proportion had a VCPR with a technical services or consulting veterinarian (4.6%; *n* = 65; CI: 1.45,13.72). Three dairies (4.6%; *n* = 65; CI: 1.45,13.72) reported having no VCPR; among these dairies, one was an organic dairy and the remaining two showed evidence of working with a local veterinarian or having a written VCPR which indicated that their response of not having a VCPR was in error or the question was misunderstood. The nature of the VCPR included a written signed agreement (47.5%; *n* = 61; CI: 38.08,60.32), verbal agreement (42.6%; *n* = 61; CI: 30.59,55.6) or being assumed based on veterinary care provided (14.8%; *n* = 61; CI: 7.71, 26.39) or based on a longtime acquaintance between the veterinarian and the producer (1.6%; *n* = 61; CI: 0.22, 11.3). All respondent calf ranches reported having a VCPR which was either written (2 out of 5), verbal (2 out of 5) or not discussed (1 out of 5).

#### Decision Making on Antimicrobial Drugs Purchased and Used on Farms

The higher proportion of respondents reported that owners made decisions for purchase and stocking of AMD added to feed or water on the dairy (69.4%, *n* = 63), compared to the veterinarian (49.2%; *n* = 63), herd manager (25.4%, *n* = 63), calf manager (20.6%, *n* = 63) and the nutritionist (3.2%, *n* = 63) (Table 8). A similar pattern was reported in the decision making to purchase injectable or oral AMD. The calf manager, however, was more commonly the decision maker to treat preweaned calves on their 1st day of illness compared to the veterinarian. The information sources that guided the producer's decision in treating preweaned calves were mainly the veterinarians (84.6%, *n* = 65), previous experience with the drug (66.2%, *n* = 65), pharmaceutical company representative (32.3%, *n* = 65), or product label (27.7%, *n* = 65). Other minor sources of information on AMD included magazines, journals, promotional materials, other producers, local/national meeting, and online materials. Cooperative extension and FARAD (Food Animal Residue Avoidance Databank) were not among the information sources reported (Table 9).

**Table 8:** Decision making on antimicrobial drugs (AMD) purchased and used to treat sick calves on California dairies.

**Person making decision**	**Percent**	**SE**	***N***	**95% Confidence limits**	
				**Lower**	**Upper**
Purchase of AMD added in feed^*^					
Owner	69.35	5.9	63	56.5	79.77
Veterinarian	49.2	6.35	63	36.83	61.68
Herd manager	25.4	5.53	63	15.97	37.89
Calf manager	20.63	5.14	63	12.19	32.75
Nutritionist	3.17	2.22	63	0.76	12.24
Purchase Injectable or oral AMD^*^					
Owner	65.67	5.84	67	53.27	76.25
Veterinarian	46.27	6.14	67	34.47	58.5
Herd manager	31.34	5.71	67	21.18	43.68
Calf manager	25.37	5.36	67	16.2	37.42
Nutritionist	1.49	1.49	67	0.2	10.31
Other^a^	1.49	1.49	67	0.2	10.31
Treatment of preweaned calves on 1st day of illness^*^					
Owner	50.06	6.16	66	43.65	67.76
Veterinarian	28.79	5.62	66	18.96	41.13
Herd manager	21.21	5.07	66	12.81	33.04
Calf manager	45.45	6.18	66	33.63	57.81
Nutritionist	0				
Other^b^	1.52	1.52	66	0.2	10.47

*The dairy owner and the veterinarian were reported to play a major role in deciding on which AMD are purchased and stocked on the dairy, as well as the choice of the AMD used to treat calves on the 1st day of illness. N, total number of respondents who answered the specific question*.

* *Some respondents chose more than one option*.

a *Son of calf-feeder*.

b *Calf-feeder*.

**Table 9:** Information sources for antimicrobial drugs used to treat preweaned calves on California dairies.

	**Percent**	**SE**	***N***	**95% Confidence limits**	
				**Lower**	**Upper**
Previous experience	66.2	5.91	65	53.55	76.81
Product label	27.7	5.59	65	17.98	40.09
Sales representative	32.3	5.85	65	21.86	44.88
Websites	1.5	1.54	65	0.2	10.62
Promotional materials	9.2	3.62	65	4.11	19.42
FARAD (Food Animal Residue Avoidance Databank)	0	–	–	–	–
Cooperative extension	0	–	–	–	–
Veterinarian	84.6	4.51	65	73.35	91.66
Other producers	16.9	4.69	65	9.47	28.39
Magazines and Journals	12.3	4.1	65	6.16	23.09
Local/national meetings	3.1	2.16	65	0.74	11.88
Other^a^	12.3	4.1	65	6.16	23.09

*The respondents were reportedly reliant on the veterinarian and previous experience with a drug as sources of information on AMD used to treat calves. The respondents typically relied on more than one information source. N, number of respondents who answered the questions*.

a *Do not use antibiotics*.

The majority of dairy producers who responded to the survey reported that that they consulted veterinarians on disease management decisions (96.8%). Similarly, veterinarians were the main prescribers for AMD used in feed or water (71.4%), besides other personnel such as nutritionists and pharmaceutical veterinarians and sales representatives. Similarly, all the calf ranches consulted the veterinarians on disease management and antimicrobial use. Table 10 summarizes the role of veterinarians and other livestock health professionals in providing consultancy services on animal disease management and prescription for AMD use.

**Table 10:** Consultation and prescription of antimicrobial drugs used to manage diseases in preweaned calves on California dairies.

**Decision on disease management**	**Percent**	**SE**	***N***	**95% CI**	
				**Lower**	**Upper**
**Producers' consultant**					
Veterinarian	96.8	2.3	62	87.6	99.2
Nutritionist	17.7	4.9	62	9.9	29.7
Pharmaceutical Co. vet/nutritionist	12.9	4.3	62	6.5	24.1
Pharmaceutical Co. Sales rep.	12.9	4.3	62	6.5	24.1
Other^a^	1.6	1.6	62	0.2	11.1
**Who prescribes (or authorizes) antimicrobials in feed or water**					
Veterinarian	71.4	6.1	56	57.9	82
Nutritionist	7.1	3.5	56	2.6	18
Pharmaceutical Co. vet/nutritionist	0		56		
Pharmaceutical Co. Sales rep.	1.8	1.8	56	0.2	12.3
Other^b^	16.1	5	56	8.4	28.6

*The respondents mainly consulted the veterinarians on the type of AMD used to treat preweaned dairy calves. Similarly, the veterinarians were the principal personnel prescribing AMD for dairy calves. N, number of respondents who answered the question*.

a *Milk replacer and grain company consultant*.

b *Used containing antimicrobials (supplied by company)*.

### Health Management Practices

Health management practices explored included colostrum management and vaccination practices. Most of the responding premises did not heat-treat colostrum fed to calves (87.1%; *n* = 139; CI: 80.28, 91.73). Intranasal vaccination against respiratory pathogens was the most used form of vaccine delivery in calves (77.6%; *n* = 58; 64.70, 86.74), administered at a mean age of 5.5 days (*n* = 41); other vaccine types reported included modified live vaccines against pneumonia or diarrhea causing pathogens (52.5%; *n* = 58; CI: 39.54, 65.21) administered at a mean age of 35.5 days (*n* = 31) days, and killed vaccines against pneumonia or diarrhea pathogens (20.7%; *n* = 58; CI: 11.94, 33.43) given at mean age of 19.3 days (*n* = 15).

### Multiple Factor Analysis

Multiple factor analysis of responses to 66 survey questions identified six components from which 25 variables explained most of the variability in the survey responses (Table 11). The first three dimensions described 21.46% of the variability in the data set. The first dimension explained 9.75% of the variability with most of the variability (65.23%) explained by use of diagnostics to guide treatment with AMD (18.60%), the source of information and decision on AMD (17.95%), treatment protocols and records (17.64%), and the common drugs used for treatment of diarrhea and pneumonia (11.06%). The second dimension explained 6.56% of the variability with calf management practices explaining 16.70% of the variability with the highest correlation 0.642 to pasteurization of milk (yes/no) for pre-weaned calves. The highest variation explained on the third dimension (37.13%) was related to the changes made in AMD use on dairies post-VFD final rule change, with high correlation determined for changes in AMD administered to pre-weaned calves in grain, water, injectables, and milk or milk replacer (0.670, 0.633, 0.629, 0.623, respectively).

**Table 11:** Component variables explaining variability in antimicrobial drugs (AMD) in preweaned dairy calves on California dairies.

**Identified components**	**Variation Percent (%)**	**Component variables**	**Correlation**
1. Change in the use of antimicrobial drugs following	37.13	Antimicrobials used on solid feed	0.670
the implementation of Veterinary Feed Directive		Antimicrobials used in water	0.633
(VFD) in 2017		Injectable antimicrobials	0.629
		Antimicrobials used in milk or milk replacer	0.623
		Producer considers AMD importance in raising calves	0.521
		Antibiotic drug costs since VFD final rule	0.477
		Started use of alternatives to antimicrobials	0.475
2. Use of diagnostics to guide treatment decision	18.60	Submission of calves to diagnostic labs	0.674
		Use of diagnostic techniques to guide treatment	0.491
		Frequency of animal health monitoring by veterinarian	0.468
3. Source of information and decision on AMD for	17.95	Decision to use injectable or oral antimicrobials (Vet/Non-vet)^a^	0.618
feed, milk, water, oral, and injectable		Source of information on antimicrobials (Vet/ Non-vet)^a^	0.582
		Decision to use any antimicrobials (Vet/Non-vet)^a^	0.573
		Method of estimating the drug dosage (Vet/Non-vet)^a^	0.552
		Decision to treat on 1st day of illness (Vet/Non-vet)^a^	0.548
		Decision on second choice antimicrobial (Vet/Non-vet)^a^	0.510
4. Antimicrobial use protocols and records	17.64	Estimation treatment duration for any antimicrobial (Label/Others)	0.613
		Tracked treatment information (Date/Route/Dose/None)	0.574
		Estimation of treatment duration for injectable antimicrobials (Label/Others)	0.506
		Tracked antibiotic withdrawal interval (Yes/No)	0.446
		Treatment protocols components (Vaccinations/Disease Definition/Treatment)	0.432
		Method of tracking treatments (Computer/Paper/Hutch/Memory/Chalk)	0.436
		Keep drug inventory log (Yes/No)	0.425
5. Common drugs to treat pneumonia and diarrhea	11.06	First choice antibiotic for treatment of Pneumonia (1-florfenicol, 2- 3rd generation cephalosporins, 3-Tulathromycin, Gamithromycin and Tildipirosin, 4-Oxytetracycline, 5-Pencillin and Ampicillin, 6-Tylosin, 7-Enrofloxacin)	0.511
6. Calf feed management	16.70	Milk fed to preweaned calves (Pasteurized/ Not)	0.642

*The multiple factor analysis identified six components with 25 component variables with correlation >0.40 at the first three dimensions. The first three dimensions of MFA explained 21.46% of the variability (dimension 1 = 9.75%; dimension 2 = 6.56%; dimension 3 = 5.15%)*.

a *Varaibility due to veterinarian making decision compared to non-veterinarian*.

## Discussion

The survey response rate of 12% in this study was comparable to the 15% response outcome from a previous mailed survey of the same demographics (37). The mean characteristics of the respondent dairies (herd size, rolling herd average, and breed composition) mirrored the state averages for the year 2016 (38), indicating the respondents were representative sample of the dairy farmers in the area. Similarly, majority of the respondents were from GSCA which has the highest number of dairy farms within the state. Most of the producers (82.7%) were aware of the recently implemented regulatory changes in the VFD rules which could have been due to extensive awareness campaigns at both the national and state levels through online resources, fact sheets, and other materials as well as workshop presentations at producer and veterinary meetings. Use of AMD was generally perceived as important in raising calves and the respondents thought calf health and welfare would be negatively affected if AMD were no longer available. The same opinion was expressed among dairy farmers in Tennessee during focus group discussions on the impact of VFD changes on the ability of producers to prevent disease on their herds; these producers indicated that the VFD regulation had limited access to essential AMD which led to increased disease occurrence and deaths particularly among calves, and reduced growth rate (39). It is worth noting that the outcome of this study could have been influenced by the negative perception of surveyed producers toward regulatory changes and may not correlate to actual increase in disease occurrences. Comparatively, the European ban on the use of growth-promoting antimicrobials was mainly associated with increased early postweaning diarrhea in piglets and enteritis in broiler chicken, while minimal or no negative clinical effects on the ban was reported in other animal species (40–42).The increased disease burden in affected animal species resulted in increased use of antimicrobials for treatment and prevention, a challenge that was later addressed by improvements in animal health management and housing (41).

Among the five key AMD stewardship practices stated in the survey (judicious AMD use, good record keeping, having a valid VCPR, observing withdrawal period and using alternatives to AMD), the respondents ranked judicious drug use (appropriate drug, dose, route, and duration of use) as the most important, and the use of alternatives to AMD as the least important. This finding indicates that the respondents appreciated the concept of judicious drug use and highlights other aspects of stewardship, such as disease prevention and the use of alternatives to AMD, which should be the focus of future outreach efforts. Having a valid VCPR was ranked 4th (low), although up to 90% of the respondents had a valid VCPR. It is possible the respondents considered having a valid VCPR to be a regulatory requirement for access to AMDs, rather than a stewardship practice. Most of the respondents reported no change in the use of AMD post-VFD rule changes, although a small proportion indicated a decrease in the use of AMD, primarily for those administered in milk fed to calves. The limited changes following the implementation of the VFD final rule may be attributed to the short time lapse (6 months) between the implementation date and administration of this survey. It is worthwhile though that the key change noted among a few respondents was a reduction in use of antimicrobials. However, the producers that reported decreased use of OTC drugs due to the VFD requirement also indicated a resultant increase in calf mortality. It is possible that respondents who reported increased calf mortality post-VFD were partly reliant on AMD prophylaxis for disease prevention prior to VFD implementation. In addition, the survey respondents could have been biased in their responses by a perceived negative effect of the regulatory changes on AMD use. Opportunities exist to improve and manage calf health through vaccination, use of diagnostics to guide treatment decisions, and use of supportive therapy when AMD use is not justified, such as cases of viral enteritis. Indeed, very respondents reported use of salt solutions as the first-choice treatment for diarrhea, and less than one-third of the respondents either reported submission of calves for diagnosis or use of other diagnostic methods to guide treatment of preweaned calves. Furthermore, there is need for future on-farm studies to generate data on-farm changes in AMD use in preweaned calves post-VFD and the associated impact on calf health and mortality. In addition, longitudinal studies that investigate implementation of stewardships practices that reduce unnecessary use of AMDs and the animal health and welfare outcomes are needed.

Feeding calves colostrum or non-saleable (waste) milk containing AMD residues, as reported by most respondents, constituted potential sources for indirect exposure of preweaned calves to AMD. Previous studies on California dairies reported detectable concentrations of at least one AMD compound in 15 out of 25 waste milk samples tested (43). The presence of AMD in waste milk could potentially contribute to development of AMR (2). Since waste milk is a valuable feed source for calves (44), stewardship efforts should focus on strategies to reduce residues in waste milk to mitigate the potential risk of AMR development. Although most dairies pasteurized waste milk prior to feeding to calves, pasteurization is not effective in removing residues, and calves fed pasteurized waste milk were shown to have increased presence of AMR gut bacteria compared to calves fed milk replacers (16, 45). Recent research evidence shows that alkalinization of milk to pH 10 and spiked with ceftiofur sodium resulted in 96% degradation of the initial drug concentration (46), and is thus a potential strategy to treat waste milk before feeding to calves. Such a strategy would increase wider use of waste milk for feeding calves as a low-cost diet alternative and reduce costs associated with its disposal otherwise.

Treatment of calf diseases was the main cause of direct exposures of calves to AMD. This finding is consistent with the outcome of a previous survey of Tennessee cattle producers which showed that treatment of clinical disease and animal welfare were some of the key drivers for AMD use (23). With regards to the specific AMD types, the highest mean percentage of calves that were administered a given AMD type was reported for tetracycline and neomycin added in milk, grain, and water. In the United States, tetracycline and neomycin are among the drugs currently labeled for treatment of diarrhea (scours) (47, 48), which is the most common preweaning calfhood disease (49). Some respondents listed non-antimicrobial compounds amongst AMD administered treat preweaned calves indicating the need for awareness to correctly identify drug classes. Whereas, most of the respondents reported keeping treatment records, treatment date was the only common information recorded, and only a few respondents recorded additional information such as the drug dose, duration of treatment and route of administration. In addition, only half of the respondents used permanent computer software or paper records, the remaining respondents relied on hutch markings, memory, used chalk or did not keep records. Record keeping is one of the key elements of AMD stewardship, and future efforts to promote antimicrobial stewardship for preweaned calves should address this area.

Some of the reported uses of AMD in preweaned calves indicated usages that are not permitted by FDA. Among these uses were a few responses that indicated administration of spectinomycin or coccidiostats in milk or milk replacer. None of these products has label directions for use in milk or milk replacer, and extra-label use (ELDU) of any animal or human drug in or on animal feed is not permitted by FDA (50). By contrast, ELDU of AMD administered in water is permissible provided that the other requirements for ELDU established by FDA are properly met, which includes supervision of such use by a licensed veterinarian with a VCPR. The single reported use of enrofloxacin as an AMD choice for treatment of scours or diarrhea would be a violation of a specific FDA regulation which prohibits ELDU of this AMD in food-producing animals (51). The few responses for these uses associated with regulatory violations may be attributed to the tendency of written survey respondents to give answers they feel is correct rather than the actual practice or may represent errors by respondents in completing the survey items; otherwise, these responses indicate the ongoing need for veterinarians and producers to be vigilant of current regulations and to be in compliance with those requirements to ensure a safe food supply of animal origin.

Having a valid VCPR constitutes the regulatory and operational basis of interaction between veterinarians and their clients in provision of health care for their animals (52). Up to 90% of responding dairies reported having a VCPR. Dairies that reported not having a VCPR indicated otherwise in their responses to other questions. It is therefore most likely that 100% of the respondents had a VCPR; however, outreach efforts are further needed to inform a small percent of CA dairies on what constitutes the creation and maintenance of a VCPR with a veterinarian. Maintaining a valid VCPR allows the veterinarian to be in the best position to provide advice on AMD use decisions on farms (53). The decisions to purchase AMD for the dairy and treat sick animals with a specific AMD influence drug use on farms. In this survey, producers had a greater influence on the decision to purchase drugs, followed by veterinarians. However, the veterinarians were the major source of information that guided the producer's decisions, besides producers' experience with the drug, pharmaceutical sales representatives, and drug label information. Our finding is in concordance with previous studies that identified veterinary advice was the primary reason for choosing AMD by farmers in New Zealand (21).

Six components explained most of the variability in the survey responses. Knowledge of these components provide insights into management practices that can be the focus for stewardship interventions and outreach. The first major component identified pertained to the changes made in the use of antimicrobials following implementation of the VFD final rule, including discontinued use of one or more AMD or reduced the amount or duration of use. Such changes could have been the direct consequence of the VFD rule restricting use of AMD. Only a small proportion of respondents had started the use of alternatives to AMD, and as such future studies on stewardship should explore barriers and motivations for use of AMD alternatives. The rest of the identified components were key features of AMD management practices (disease diagnosis, AMD use practices, record keeping, information sources and decision of AMD use, and health management practices). In the health management component, heat treatment of colostrum fed to calves was the single most important variable to explain the variability between dairies. Variation in the colostrum management was possibly associated with farm size as shown in a previous survey of Pennsylvania dairy farmers in which larger farms were more likely to have the equipment for colostrum heat treatment (54).

One limitation of the current study was the small number of responses (*n* = 5) received form the calf ranch producers. Similarly, the number dairy respondents were relatively low, although the response rate was comparable to the outcome of previous survey conducted among the same demographics. The low response could be attributed to the survey fatigue and challenges inherent in the discussions and reporting of antimicrobial drug use among food animal producers in general.

## Conclusion

Following the implementation of the VFD final rule on January 1, 2017, more than one third of the producers had made changes in the use of AMD, most notably by reducing the amount or duration of use or discontinuing the use of one or more AMD added in liquid diet or on solid feeds. The limited changes noted in AMD use could have been due to the short period between the implementation of VFD and conducting the survey. Most respondents reported a greater involvement of the herd veterinarian, compared to nutritionists or pharmaceutical sales representatives, in informing producers about the use of AMDs. Whereas, most producers had knowledge of the VFD and SB 27, opportunities exist to improve AMD use practices, including record keeping, using AMD alternatives, and improved farm management practices to reduce disease burden and need for AMD use.

The current survey outcomes allow immediate assessment of the impact of VFD final rule implementation and provides baseline data for future evaluation of the impact of VFD as well as SB 27 regulatory changes. The knowledge gained from this study is a valuable resource that could guide future recommendations for best health management practices and promote antimicrobial stewardship efforts.

## Data Availability Statement

Data collected for this study is protected under California Food and Agriculture Code 14407. Requests for raw data may be made to the authors, who will consult with the California Department of Food and Agriculture (CDFA) on fulfilling the request.

## Author Contributions

SA and EO designed the study, authored the first draft of the survey questions, and conducted the survey. SA, EO, DW, TL, RP, and JA reviewed and edited the survey. EO, SA, and WE analyzed the survey data. EO wrote the first draft of the manuscript. All authors edited and approved the final manuscript.

## Conflict of Interest

JA is an employee of the California Department of Food and Agriculture. The remaining authors declare that the research was conducted in the absence of any commercial or financial relationships that could be construed as a potential conflict of interest.
