# Supplementary material for: Survey on Antimicrobial Drug Use Practices in California Preweaned Dairy Calves
Source: Front Vet Sci. 2021 Apr 22;8:636670. doi: 10.3389/fvets.2021.636670 (PMC8101284; doi:10.3389/fvets.2021.636670)
Supplement: Supplementary file 1 [file Data_Sheet_1.PDF]

Survey of Antibiotic Drug Use on California Calf Ranches

University of California Davis, School of Veterinary Medicine faculty are reaching out to CA calf ranch owners' and managers' for their experiences with antimicrobial drugs for preweaned dairy calves. **Your responses are anonymous, confidential, and will not be shared with anyone or used to identify you or your business.** Only the summarized results of this survey will be shared with the dairy industry and stake holders through meetings, and publications. Hence, your questionnaire is identified on the first page by a confidential number which will only be used to send reminders to complete and return the survey. The survey should take 20-30 minutes of your time. We suggest having your treatment information at hand while taking the survey for quick references. If you prefer to complete the survey over the phone please email us at: survey@vmtrc.ucdavis.edu and one of the survey managers will contact you at a time of your choice. Alternatively, if you prefer to complete the survey online please use the following link: ***www.vmtrc.ucdavis.edu/survey***

The word antimicrobial is a general term that includes any drug that kills or inhibits the growth of microorganisms. An antibiotic, for example penicillin, is a specific term limited to those antimicrobials naturally produced by other microorganisms. Antimicrobials include antibiotics and other classes of drugs, such as sulfonamides and quinolones, which have been chemically developed. **For the purpose of this survey, we will refer to all antimicrobial drugs regardless of their origin as antibiotics**. Anti-inflammatory drugs (e.g. Banamine®,) and other chemicals that do not have antimicrobial activity are not the target of this survey; please do not consider them when answering the survey questions.

The objectives of this survey are to identify the use, acquisition, keeping and maintenance of antibiotics used to treat preweaned calves on dairies. Our hypothesis is that antibiotic drugs play an important role in raising preweaned calves and sustain their health and welfare. We expect results of this survey to; 1) further the understanding of the industry's needs and expectations for the availability and effectiveness of antibiotics for preweaned calves, and 2) guide future recommendations and best practices. The raw data collected through this survey are confidential and are protected from requests for access or any unauthorized distribution or release as provided by section 14407 of the Food and Agricultural Code. Hence, the raw data (your information and responses) are protected and **will not be shared** with any persons or entities including your veterinarian and the funding source of this survey, the Animal Health and Food Safety Services of the California Department of Food and Agriculture. The faculty and staff of the UC Davis School of Veterinary Medicine are committed to protecting and maintaining the confidentiality of your data through all phases of data collection in addition to only reporting aggregated or summarized results.

Sincerely,

Sharif Aly	Emmanuel Okello	John Champagne
Terry Lehenbauer	Deniece Williams	Richard Pereira
