# Supplementary material for: Survey on Antimicrobial Drug Use Practices in California Preweaned Dairy Calves
Source: Front Vet Sci. 2021 Apr 22;8:636670. doi: 10.3389/fvets.2021.636670 (PMC8101284; doi:10.3389/fvets.2021.636670)
Supplement: Supplementary file 2 [file Data_Sheet_2.PDF]

Date: ……/………/……… Confidential #:

Survey of Antibiotic Drug Use on California Calf Ranches

School of Veterinary Medicine, UC Davis

- *If you own and/or operate more than one calf ranch, please answer the questionnaire based on the calf ranch to which this survey was addressed.*
- *Please do not write your name anywhere on the questionnaire to maintain confidentiality.*
- *If you are not sure of an answer, please give us your best estimate.*
- *Unless requested to check all that apply, please mark only one answer per question.*
- *Try to answer all the questions, unless prompted to skip a section.*
- *Please return the completed survey in the self-addressed stamped envelope.*

Thank you for your participation in this confidential survey

QUESTIONS IN THIS SURVEY REFER TO THE CURRENT YEAR OF 2017

SECTION 1: HERD INFORMATION

1. What is your role/position in managing the calf ranch? ***Please check all applicable boxes.***

   ☐ Owner ☐ Herd manager
   ☐ Calf Manager ☐ Calf Feeder(s)
   ☐ Other, please specify_______________

2. In which county is your calf ranch located? ______________________________________

3. What is the average number of preweaned calves arriving on your premise weekly? ____________

4. What are the predominant breed(s) in your herd? ***Please check all applicable boxes and write the approximate percent***

   ☐ Holstein ______ % ☐ Crossbred _____ %
   ☐ Jersey ______ % ☐ Other _____ %

5. Which calf age groups do you raise? Please check all applicable boxes.

   ☐ Preweaned heifers & bulls ☐ Preweaned heifers only ☐ Preweaned bulls only
   ☐ Postweaned heifers & bulls ☐ Postweaned heifers only ☐ Postweaned bulls only
   ☐ Other, ***please describe:***____

6. Do you participate in animal welfare assessment programs for calves?

   ☐ No ☐ Yes, ***please specify:*** _______________________________

SECTION 2: PRE-WEANED CALF MANAGEMENT PRACTICES

1. What is the average age of calves when they arrive on your premise? _______________Days

2. Are calves fed colostrum at the **dairy of origin**?

- ☐ No (Proceed to question 5)
- ☐ Yes, all calves. ***Please state number of feedings***: ___________________________________
- ☐ Yes, some calves. ***Please state number of feedings:*** ________________________________
- ☐ I don't know

3. Approximately what proportion of the colostrum fed to newborn calves at the **dairy of origin** is from each of the following sources?

☐ Individual cow_____ % ☐ Colostrum replacer _____ % ☐ I don't know

☐ Pooled _____ % ☐ Nurse from dam _____ %

4. Do you feed colostrum to calves upon arrival **on your calf ranch**?

- ☐ No
- ☐ *Yes,* ***please state number of feedings/times***:_____________________________________

5. Approximately what proportion of the colostrum fed to newborn calves **on your calf ranch** is from each of the following sources?

☐ Individual cow _____ % ☐ Colostrum replacer _____ % ☐ I don't know

☐ Pooled _____ % ☐ Nurse from dam _____ %

6. Is colostrum fed to calves heat-treated and/or pasteurized?

- ☐ No
- ☐ Yes, ***please describe (temperature/duration of heat):*** ____________________________

____

7. Currently, which of the following sources of milk are fed to calves and at what age? (such as birth to 7 days; 1 to 4 weeks; 3 to 8 weeks, etc.) ***Please check all applicable boxes and circle whether age you reported is in days, weeks or months.***

☐ Non-saleable milk ("hospital milk")	Ages: _________	(days/weeks/months)
☐ Transition milk from cows 1 to 3 days in milk	Ages: _________	(days/weeks/months)
☐ Saleable bulk tank milk	Ages: _________	(days/weeks/months)
☐ Non-medicated milk replacer	Ages: _________	(days/weeks/months)
☐ Medicated milk replacer	Ages: _________	(days/weeks/months)

8. What percent of the liquid diet fed to preweaned calves is:

Non-saleable ("hospital") ____ % Milk replacer _____ %

Saleable ("bulk tank") _____ % Other _____ %, ***Please specify***:________________

9. **Before January 1, 2017**, which of the following best describes the management or health protocols regarding use of antibiotics in **milk or milk replacer** for preweaned calves:

☐ Calves were not fed antibiotics in milk or milk replacer.

☐ Calves were fed antibiotics in milk or milk replacer on a limited basis or intermittently as determined by infectious disease conditions (e.g., scours or pneumonia) and then discontinued after disease condition changed or improved.

☐ Calves were fed antibiotics in milk or milk replacer on a regular or repeated pattern of use due to ongoing infectious disease conditions or risks.

☐ Other, ***please describe:***____

____

10. **Before January 1, 2017**, which of the following best describes the management or health protocols regarding use of antibiotics in **grain or solid feed** for preweaned dairy calves (Please do not consider these antibiotics used alone in grain or solid feed to control or prevent coccidiosis: amprolium [Corid®], monensin [Rumensin®], or lasalocid [Bovatec®])

☐ Calves were not fed antibiotics in grain or solid feed.

☐ Calves were fed antibiotics in grain or solid feed on a limited basis or intermittently as determined by infectious disease conditions (e.g., scours or pneumonia) and then discontinued after disease condition changed or improved.

☐ Calves were fed antibiotics in grain or solid feed on a regular or repeated pattern of use due to ongoing infectious disease conditions or risks.

☐ Other (e.g. growth promotion), ***please describe:***____________________________________

____

11. **Since January 1st 2017**, did you make any management or protocol changes in the use of antibiotics fed to preweaned calves in milk, milk replacer, grain or solid feed?

☐ No – (No changes were made in use of antibiotics being fed to preweaned calves.)

☐ Yes – For milk or milk replacer only

☐ Yes – For grain or solid feed only

☐ Yes – For both milk or milk replacer and grain or solid feed

If Yes, please describe changes that were made in protocols or management practices for antibiotics being fed to preweaned calves:____

____

12. Is milk treated before being fed to calves? ***Please check all applicable boxes.***

☐ Yes, it is pasteurized ☐ Yes, it is treated with sorbitol

☐ No ☐ Other, ***please explain:***______________________________

____

13. Which types of calf housing styles are used on this calf ranch for preweaned calves? ***Please check all applicable boxes and circle choices provided for wood / plastic/ or metal.***

☐ Wooden "California" style hutches; Average number of calves per unit: ________________

☐ Hutch (wood / plastic / metal) with fenced (wood / plastic / metal) exercise area

☐ Hutch (wood / plastic / metal) without exercise area

☐ Group pen, range of number of calves per pen: ____________________________________

☐ Other, ***please describe***: ____

14. Which of the following vaccinations are used in preweaned calves? ***Please check all applicable boxes and specify approximate ages of calves when vaccines are administered.***

☐ **Intranasal vaccine** to prevent pneumonia in **calves** given at age:______________________

☐ **Modified live** vaccine to prevent diarrhea or pneumonia in **calves** given at age:___________

☐ **Killed vaccine** to prevent diarrhea or pneumonia in **calves** given at age: ________________

☐ **I do not know/remember**

SECTION 3: ANTIBIOTIC USE IN PRE-WEANED CALVES

1. Which of these sources do you rely on for information on antibiotics used to treat calves? ***Please check all applicable boxes.***

☐ Previous experience with the drug　☐ Veterinarian　☐ Other producers

☐ Review or refer to product drug label　☐ Magazines/Industry Trade Journals

☐ Drug company or sales representative　☐ Local/ National Meetings

☐ Websites (drug co., producer blogs, etc.)　☐ Promotional materials from drug companies

☐ FARAD (Food Animal Residue Avoidance databank)

☐ State/ County/ University cooperative extension

☐ Other, ***please specify***:____

2. Who decides which antibiotics are purchased and stocked on your calf ranch? ***Please check all applicable boxes.***

a. Antibiotics added to feed (including milk or milk replacer) and water:

☐ Owner　☐ Veterinarian

☐ Nutritionist (Non-veterinarian)　☐ Calf manager

☐ Other***, please specify:*** ____

b. Injectable or oral (bolus/drench) administered antibiotics

☐ Owner　☐ Veterinarian

☐ Nutritionist (Non-veterinarian)　☐ Calf manager

☐ Other***, please specify:*** ____

3. Who decides which antibiotic is used to treat preweaned calves at first day of illness?
Please check all applicable boxes.

☐ Owner ☐ Veterinarian ☐ Nutritionist (Non-veterinarian) ☐ Calf manager,

☐ Other, ***please specify:*** ____

4. Do you have treatment or health protocols (written/ computerized) for preweaned calves?

☐ No, ***please proceed to question 5***

☐ Yes; Who developed the protocols? ____

a. What do the protocols include? (***mark all that apply***)

☐ Vaccinations

☐ Disease definitions

☐ Disease-specific treatments; If checked which of the following are included?

☐ Drug dosage (based on weight, age, or specified amount of drug to be given)

☐ Duration (such as number of times or days of treatment)

☐ Withdrawal interval or withholding time before marketing

☐ Not sure about protocol details

☐ Other information about protocols:______________________________

b. Who has access to these protocols: (***mark all that apply***)

☐ Owner ☐ Calf Manager ☐ Veterinarian

☐ Herd Manager ☐ Calf feeder(s) ☐ Calf treatment crew

☐ Office Staff ☐ Nutritionist(Non-vet) ☐ I don't know

☐ Other***, please specify:*** ____

c. How often are these protocols re-viewed and revised?

☐ Once to twice a year ☐ Every few years ☐ I don't know

☐ Other, ***please specify***:____

5. Do you keep or maintain a drug inventory log on your calf ranch? ☐Yes ☐ No

6. Which of the following drug-related information do you track or follow on your calf ranch?
Please check all applicable boxes.

☐ Name of drug ☐ Quantity on hand ☐ Date of purchase

☐ Manufacturer ☐ Drug Supplier/Source ☐ Cost of Drug

☐ Drug expiration date ☐ None of the above

☐ Other***, please specify***: ____

7. How would you say antibiotics for preweaned calves are most commonly used on your farm? ***Please check all applicable boxes.***

☐ Treat sick animals ☐ Control the spread of an ongoing disease in the herd

☐ Prevent disease in calves at high risk to a specific disease

☐ Other***, please specify:*** ____

8. How do you usually estimate the antibiotic dosage for preweaned calves?
Please check all applicable boxes

- ☐ Estimate animal weight and use label dosage
- ☐ Estimate animal weight and use a higher (or lower) dosage based on experience or previous treatment outcomes
- ☐ Estimate animal weight and use a higher (or lower) dosage as authorized by veterinarian
- ☐ Use a standard dose by category of animal, such as calf, heifer or cow
- ☐ Based on how sick the animal appears
- ☐ Use different approaches for different drugs and/or diseases
- ☐ Other, ***please specify:*** ____

9. How do you usually estimate the treatment duration for preweaned calf with antibiotics:

a. Added to feed (including milk) or water? ***Please check all applicable boxes.***

- ☐ Follow label treatment duration instructions
- ☐ Stop the use earlier if animals seem to be cured (no more clinical signs)
- ☐ Extend the use if animals still seem to be sick (still have clinical signs)
- ☐ Based on previous results using the drug on the farm
- ☐ Use different approaches for different drugs and/or disease
- ☐ Other, ***please specify:*** ____

b. Administered via injection or orally (bolus/drench)? ***Please check all applicable boxes.***

- ☐ Follow label treatment duration instructions
- ☐ Stop the use earlier if animals seem to be cured (no more clinical signs)
- ☐ Extend the use if animals still seem to be sick (still have clinical signs)
- ☐ Based on previous results using the drug on the farm
- ☐ Use different approaches for different drugs and/or disease
- ☐ Other***, please specify:***____

10. How do you base your decision for selecting a second antibiotic drug to treat a sick animal if the first treatment was not satisfactory? ***Please check all applicable boxes***

- ☐ Based on bacterial culture and antibiotic sensitivity results from a laboratory
- ☐ Talk with the veterinarian
- ☐ Follow information outlined in the farm's protocol for that disease or condition
- ☐ Based on previous results using the drug on the farm
- ☐ Other***, please specify***: ____

____

11. Which antibiotic treatment information do you track or record for preweaned calves on your calf ranch? ***Please check all applicable boxes***

☐ Date of treatment ☐ Dose ☐ Route

☐ None of the above ☐ Other***, please specify:*** ______________________________

12. How do you track antibiotic treatments given or administered to preweaned calves on your calf ranch? ***Please check all applicable boxes***

☐ Computer software, please specify: ____

☐ Paper records kept in barn or office

☐ Memory

☐ Treatment record/card attached to, or markings/clips on calf/hutch or housing unit

☐ Other***, please specify:*** ____

13. Do you keep track of antibiotic withdrawal intervals (withholding periods) for treated calves?

☐ No

☐ Yes. ***If Yes, please check all applicable boxes:***

☐ Marking calf's hutch ☐ Paper records ☐ Memory

☐ Computer software, ***please specify:*** _____________________________________

☐ Other***, please specify:*** ____

14. **In 2017**, what percent of preweaned calves received the following medications for any duration from birth to weaning? ***Example, enter 100% if all or 0% if drug not used***

**Medication**	**% Calves treated in:**		
	**Liquid feed (milk or milk replacer)**	**Solid feed (grain)**	**Water**
Biosol, Neo-Sol, or Neomycin Liquid (neomycin sulfate)			
Aureomycin® (chlortetracycline)			
Neo-Terramycin® 100/100 (Neomycin-Oxytetracycline)			
Linco-Spectin 100, L-S 50 (lincomycin-spectinomycin)			
Spectam, (spectinomycin)			
TM® Crumbles, Terramycin® 200, Oxytet, Terra-Vet (oxytetracycline)			
SMZ-Med® 454, Sulmet® (sulfamethazine)			
BMD® Soluble, Albac® 50 Granular, (bacitracin)			
Coccidiostats such as Corid®, Deccox®, Rumensin®, Bovatec®			
Other (specify):			

15. Please complete the table below with regards to antibiotics used to individually treat preweaned calf diseases or disorders on your calf ranch **since January 1st 2017**:

**Disease or disorder**	**First choice antibiotic**	**Second choice antibiotic**
**Respiratory** (including head and ear tilt)	Drug:______________________ __________________________ Used per label: ☐ Yes ☐ Don't know, not sure ☐ No, ***please describe:*** __________________________ __________________________	Drug:______________________ __________________________ Used per label: ☐ Yes ☐ Don't know, not sure ☐ No, ***please describe:*** __________________________ __________________________
**Diarrhea or other digestive conditions**	Drug:______________________ __________________________ Used per label: ☐ Yes ☐ Don't know, not sure ☐ No, ***please describe:*** __________________________ __________________________	Drug:______________________ __________________________ Used per label: ☐ Yes ☐ Don't know, not sure ☐ No, ***please describe:*** __________________________ __________________________
**Other, specify:** ______________ ______________ ________	Drug:______________________ __________________________ Used per label: ☐ Yes ☐ Don't know, not sure ☐ No, ***please describe:*** __________________________ __________________________	Drug:______________________ __________________________ Used per label: ☐ Yes ☐ Don't know, not sure ☐ No, ***please describe:*** __________________________ __________________________

16. Were any antibiotics used in off-label fashion (different dose, route, duration or disease indication for use, for example) in 2017?

☐ Yes ☐ No ☐ I don't know

17. Have you submitted any calves to a diagnostic lab for diagnosis of infectious diseases in 2017?

☐ Yes ☐ No ☐ I don't know

18. Have any other diagnostic techniques or procedures such as culture, auscultation (listening to a calf's chest with a stethoscope), lung ultrasound, etc. been used to guide treatment decision with antibiotics for preweaned calves?

☐ Yes ☐ No ☐ I don't know

19. Relating to preweaned calf diseases:

a. Who do you often consult with? ***Please check all applicable boxes***

☐ Veterinarian

☐ Nutritionist (Non-veterinarian)

☐ Pharmaceutical company veterinarian/consultant

☐ Pharmaceutical company sales representative

☐ Other, ***please describe:***____

b. Who prescribes antibiotics in feed (including milk) or water? ***Please check all applicable boxes***

☐ Veterinarian

☐ Nutritionist (Non-veterinarian)

☐ Pharmaceutical company veterinarian/consultant

☐ Pharmaceutical company sales representative

☐ Other, ***please describe:***____

20. Do you have a veterinarian-client-patient relationship (VCPR) for this calf ranch?

☐ No, ***please skip to question 23***

☐ Yes

If Yes, which choice best describes your veterinarian

☐ A private or local veterinarian or clinic

☐ A technical services veterinarian or a consulting veterinarian

☐ Other, ***please describe:***____

21. Your VCPR can best be described as:

☐ A written agreement signed by you and your veterinarian

☐ A verbal agreement between you and your veterinarian

☐ A VCPR was not formally discussed but I consider that I have one based on the veterinary care my calves receive through my veterinarian

☐ Other, ***please describe:***____

____

22. How often does your prescribing veterinarian observe, monitor, or discuss with you the health of your preweaned calves?

☐ Regular intervals, every _______ days/weeks/months. ***Please circle the applicable interval***

☐ As needed ☐ Other, ***please describe:*** ____

23. ***Please do not consider monensin (Rumensin®), lasalocid (Bovatec®), amprolium (Corid®), or decoquinate (Deccox®):*** Since implementation of FDA's final rule of the Veterinary Feed Directive in January 2017, what changes did you make for preweaned calves compared to 2016 with regards to:

a. **Antibiotics used in milk or milk replacer:**

☐ No changes have been made.

☐ Same antibiotics are being used but the amount or duration **increased**

☐ Same antibiotics are being used but the amount or duration **decreased**

☐ One or more antibiotics have been discontinued.

☐ One or more antibiotics have been added.

☐ Other, ***please describe:*** ____

b. **Antibiotics used in grain or solid feed:**

☐ No changes have been made.

☐ Same antibiotics are being used but the amount or duration **increased**

☐ Same antibiotics are being used but the amount or duration **decreased**

☐ One or more antibiotics have been discontinued.

☐ One or more antibiotics have been added.

☐ Other, ***please describe:*** ____

c. **Antibiotics used in water:**

☐ No changes have been made.

☐ Same antibiotics are being used but the amount or duration **increased**

☐ Same antibiotics are being used but the amount or duration **decreased**

☐ One or more antibiotics have been discontinued.

☐ One or more antibiotics have been added.

☐ Other, ***please describe:*** ____

d. **Injectable antibiotics in preweaned calves:**

☐ No changes have been made.

☐ Same antibiotics are being used but the amount or duration **increased**

☐ Same antibiotics are being used but the amount or duration **decreased**

☐ One or more antibiotics have been discontinued.

☐ One or more antibiotics have been added.

☐ Other, ***please describe:*** ____

24. ***Since*** implementation of FDA's final rule of the Veterinary Feed Directive in January of 2017, has the use of over-the-counter (OTC) antibiotics labelled for feed that do not fall under the VFD (example, Bacitracin) changed and if so, how?

☐ Increased, ***please specify:*** ____

☐ Decreased, ***please specify:*** ____

☐ I do not know

25. Since implementation of FDA's final rule of the Veterinary Feed Directive in January of 2017, have you used alternatives to antibiotics? ***Please check all applicable boxes:***

☐ Vitamins, ***please explain:*** ____

☐ Minerals, ***please explain:*** ____

☐ Herbal remedies , ***please explain:*** ______________________________________

☐ Pathogen specific antibodies commonly derived from eggs

☐ Other***, please explain:*** ____

____

26. Since implementation of FDA's final rule of the Veterinary Feed Directive on January 1st 2017, how would you describe your antibiotics drug costs related to preweaned calves?

☐ Increased ☐ Decreased ☐ No change

27. Please describe the importance of antibiotics in raising healthy preweaned calves on your calf ranch by identifying areas that may be affected if you stopped using antibiotics: ***Please check all that apply.***

☐ Prevalence of calf diseases will increase

☐ Calf welfare will be affected

☐ Calf growth or performance will decrease

☐ No effect or no difference

☐ Organic calf ranch; antibiotics not used regardless of importance

☐ Other, ***please describe:*** ____

____

28. Please rank the importance of antibiotics in raising preweaned calves on your calf ranch: ***Please choose only one response.***

☐ Extremely important, raising calves without antibiotics is very challenging or not possible.

☐ Important, raising calves without antibiotics will lead to decrease in health, welfare and growth.

☐ Somewhat important, can raise calves without antibiotics but will require modifications in calf management including alternatives such as more vaccines or supplements.

☐ Not important, can raise calves without antibiotics with minimal or no modifications.

☐ Organic calf ranch; antibiotics not used regardless of importance

SECTION 4: CURRENT REGULATIONS

1. Below is an alphabetical list of antibiotic drug use stewardship practices. ***Please rank these practices from 1 (most important) to 5 (least important):***

   ______ Administration of the appropriate antibiotic drug, dose, route and duration

   ______ Good record keeping on treatments and treatment dates

   ______ Having a current Veterinary Client Patient Relationship (VCPR)

   ______ Observing withdrawal periods and drug residue avoidance

   ______ Pursuing other alternatives to antibiotic drugs (e.g. vaccines, supplements)

2. Are you aware that beginning on January 1, 2018, all uses of antibiotics currently sold over-the-counter, including injectable antibiotics such as Penicillin Injectable, Liquamycin® LA 200 (oxytetracycline), and Tylan® Injection (tylosin), and boluses, such as Supra Sulfa® III or Sustain III (sulfamethazine), will require a veterinary prescription and will no longer be sold over-the-counter in California?

   ☐ Yes ☐ No

3. For preweaned calves, are you currently treating calves with any injectable antibiotics or any antibiotic boluses that are purchased over-the-counter (OTC) and are being used according to label directions without any additional changes in dosages, routes, or durations of treatment?

   ☐ Yes, OTC antibiotics are being used according to label directions

   ☐ No, OTC antibiotics are being used in ways that are different from label directions

   ☐ No, OTC antibiotics are not being used

   ☐ Do not know, not sure

You have reached the end of the questionnaire. If you have any additional comments about antibiotic use in preweaned calves or other dairy cattle please share them in the space below.

Thank you so much for your cooperation.
